# Capturing the wisdom of the crowd: health professions’ educators meet at a virtual world café

**DOI:** 10.1007/s40037-020-00623-y

**Published:** 2020-10-13

**Authors:** Judy McKimm, Subha Ramani, Rashmi A. Kusurkar, Alice Fornari, Vishna Devi Nadarajah, Harish Thampy, Helena P. Filipe, Elizabeth K. Kachur, Richard Hays

**Affiliations:** 1 Swansea University Medical School, Swansea, Wales UK; 2 Department of Medicine, Brigham and Women’s Hospital and Harvard Medical School, Boston, USA; 3 Faculty of Medicine, Vrije University Amsterdam, Amsterdam, The Netherlands; 4 Faculty Development, Northwell Health System, New York, USA; 5 International Medical University, Kuala Lumpur, Malaysia; 6 University of Manchester, Faculty of Biology, Medicine and Health, UK; 7 College of Ophthalmology and Department of Medical Education, Faculty of Medicine, University of Lisbon, Lisbon, Portugal; 8 Medical Education Development, Global Consulting, New York, USA; 9 James Cook University, Queensland, Australia

**Keywords:** Virtual, Online, Speed mentoring, Faculty development, World café

## Abstract

**Background:**

Conversations about educational challenges and potential solutions among a globally and culturally diverse group of health professions’ educators can facilitate identity formation, mentoring relationships and professional network building. The COVID-19 pandemic has made it even more important to co-create and disseminate knowledge, specifically regarding online and flexible learning formats.

**Approach:**

Based on the principles of social learning, we combined speed mentoring and world café formats to offer a virtual Zoom™ workshop, with large and small group discussions, to reach health professions’ educators across the globe. The goal was to establish a psychologically safe space for dialogue regarding adaptation to online teaching-learning formats.

**Evaluation:**

We aimed to establish psychological safety to stimulate thought-provoking discussions within the various small groups and obtain valuable contributions from participants. From these conversations, we were able to formulate ‘hot tips’ on how to adapt to (sometimes new) online teaching-learning formats while nurturing teacher and student wellbeing.

**Reflection:**

Through this virtual workshop we realized that despite contextual differences, many challenges are common worldwide. We experienced technological difficulties during the session, which needed rapid adaptation by the organising team. We encouraged, but did not pressure, participants to use video and audio during breakout discussions as we wanted them to feel safe and comfortable. The large audience size and different time zones were challenging; therefore, leadership had to be resilient and focussed. Although this virtual format was triggered by the pandemic, the format can be continued in the future to discuss other relevant global education topics.

## Background and need for innovation

Health professions’ educators (HPEs) greatly benefit from mentorship and professional connections across geographical boundaries to reflect on common educational challenges and share ideas and solutions. A speed mentoring workshop, offered at the 2019 Association of Medical Education in Europe (AMEE) conference, provided participants with an opportunity to engage in discussions with educational leaders and peers on a variety of educational topics [1]. The COVID-19 pandemic accelerated the need for global conversations, specifically the design, implementation, and evaluation of educational initiatives that effectively promote online learning [2, 3]. The HPE community has responded by organising a range of local, regional and international online faculty development initiatives. Yet, few venues promote free exchange of ideas and sharing of success and failures in a psychologically safe environment. The team that organised the AMEE speed mentoring session believed that it would be valuable to organise interactive webinars in a non-hierarchical format, prioritising outcomes such as peer-peer learning and fostering global communities of practice (CoPs) [4].

First described by cognitive anthropologist Jean Lave and educational theorist Etienne Wenger, CoPs can be formed informally or formally based on shared professional interests [5, 6]. Virtual CoPs have the advantage of being unrestricted by geographical boundaries [7]. Such professional connections can contribute to identity formation, knowledge-sharing, mentoring relationships, and professional development [8]. CoPs are particularly important for HPEs during the COVID-19 pandemic, which forced a rapid transition to virtual educational formats across the educational continuum, leaving teachers and learners alike scrambling to learn new best practices [2]. Across the globe, HPEs and their learners are making rapid, sometimes instantaneous, adaptations to teaching and learning, whilst facing severe uncertainty. The AMEE speed mentoring team organised a virtual ‘world café’ workshop in April 2020, modelled on the in-person workshop at the 2019 AMEE meeting [1]. This article describes the innovation and lessons learned [4].

## Goal of the innovation

Recognising that educators at all stages can provide insights into the challenges of virtual teaching and learning, the key goals of this workshop were *to create a psychologically safe space in which a global and culturally diverse group of HPEs could have interactive discussions and feel supported in their efforts to design new educational initiatives *[9]. The session blended elements of speed mentoring and world café conversations where educators could interact with each other and potentially widen their networks [10–12].

Speed mentoring, an adaptation from the concept of speed dating, was originally intended for professionals to quickly meet several potential mentors and gauge their expertise, interests and compatibility [13]. This initial contact may end with the one-time conversation or lead to longer mentoring relationships [11, 12]. Speed mentoring has evolved into a distinct one-time mentoring format at institutions, professional meetings and conferences [14, 15]. World café is a flexible format that encourages conversations among small groups, with individuals moving at allotted times to a different table to engage in a new conversation (www.theworldcafe.com) [10]. Typically, a host welcomes participants, explains ground rules, facilitates and records discussions.

Speed mentoring and world café discussions can be viewed through the lens of social learning principles, integral to an era where virtual educational connections are critical [16]. In this approach, a group is defined by its purpose and shared interests, anyone interested can join, participation is by choice, and every group member can contribute knowledge and skills while learning from others. Additionally, virtual communities have the advantage of having no geographical boundaries to group membership. Professionals have a unique opportunity to interact with multiple experts and leaders across the globe, representing the spectrum of career disciplines, and capture their shared collective wisdom and varied perspectives. Leaders of such communities should possess the ability to support colleagues regardless of geographic location.

## Steps taken for development and implementation of innovation

### Workshop planning

The original AMEE speed mentoring group had transformed itself into an active community of mentors and scholars, with regular meetings, scholarly projects and a commitment to ongoing mentoring, in-person and virtual [1]. The team, self-titled *‘Mentors without Borders*’ (or MWB), comprised educational leaders who served as mentors at the AMEE workshop, and newer members who joined the community to increase geographic reach. The group followed the paradigm of one leader supported by active followers, who guided the vision, goals and strategies of the team and enabled the leader to lead effectively [17]. The team collaboratively designed the format and content for the virtual workshop, with collective sharing of ideas and delegation of roles and responsibilities using email and a series of Zoom™ meetings. The first webinar was timed to facilitate conversations relating to the abrupt transition to online learning that the vast majority of medical educators around the world were facing [3]. To recruit participants, email flyers were sent to educator groups, such as the AMEE faculty development and Harvard Macy lists, advertising the topic and goals of the session, the moderator list and a registration link. Twelve moderators and 55 participants participated in this session. The key elements of the workshop are described in detail below and in Tab. 1.

**Table 1:** Tips to organise and implement virtual interactive discussions

Stages	Steps	Sample strategies
Before the session	Form a community of mentors/leaders	Reach out to educators with a shared interest in mentoring
		Hold multiple conversations to reach consensus on the vision and strategies
		Decide on formats for virtual events- didactic vs interactive vs hybrid; formal vs informal etc.
		Respect and welcome a variety of perspectives and ideas
	Organize the virtual workshop	Decide on topics for discussion
		Discuss logistics—duration, time zone, publicity, recruitment, registration etc.
		Compose questions for polls, small group discussions
		Finalize guidelines for moderators—how to create a safe space, how to engage participants, allow participants to do most of the talking etc
		Finalize the agenda and timing for each segment of the workshop
		Do a practice run on the platform
		Assign roles to moderators, ensure back up if the session leader develops connectivity issues
		Chat moderators are critical to respond to all participant comments and questions
	Recruit participants	Recruit using known health professions educator group lists
		Decide whether to register or just advertise with a link to join the session
		Discuss whether to limit number of participants. This depends on capacity of the platform and format of the session
		Pre-assignment of breakout groups if list of participants is available ahead of time and attendance can be ensured
During the session	Welcome and introductions	Introduce each of the moderators using video and audio
		Establish ground rules
		Explore who the participants are using polls and chat-location, profession, goals for participation
		Present the agenda and goals
		If recording the session or saving chats, mention this and ensure that there are no objections
		Assurance of confidentiality of opinions
	Set the stage	Use ice-breakers-personal reflections, challenges faced/overcome, success stories
		Showcase the diversity of the group
		Demonstrate facilitative and friendly body language
		Highlight peer learning and leaders model openness to learning from others
	Small group moderation	Use trigger questions for participants to reflect on while groups are forming
		Moderators facilitate introductions in small groups‑1 personal fact and 1 professional fact
		Moderators avoid doing too much talking and ensuring participation from group members
		Request participants to share audio and video if they are comfortable, but allow chats for those who are not
	Debriefing and lessons learned	Conclude the workshop with presentation of key points raised by participants
		Moderators/experts can add other tips
		Final poll/chat on one take home point
		Thank all participants for contributing to the learning
After the session	Reflections	Session organisers meet to reflect on what went well and what needs improvement
		Reflections and lessons learned should be applied to future initiatives
		Educational initiatives, especially those are virtual and global, need a continuous quality improvement mindset

### Workshop implementation

The one-hour virtual workshop was conducted using Zoom™ which provides live video, audio and chat connectivity. We combined large and small group discussions to promote open conversations in a psychologically safe space. Participants were allocated to break out rooms for specific time slots, with pre-defined topics and trigger questions to jumpstart discussions. Two moderators facilitated each group discussion. To build safety, we used initial polls and icebreakers and the moderators encouraged members to share ideas, raise questions and provide suggestions to others’ challenges. Participants were given the choice of using audio, video or chats, based on their comfort level and internet connectivity. The moderators were international educational leaders with considerable experience in mentoring and facilitating group discussions. Regular virtual meetings and a practice session established ground rules for moderation.

### Large group introduction

The workshop began with a welcome greeting, which included advice on online etiquette, confidentiality assurance to ensure a safe and trustful educational environment and a roadmap of the session. Participants were asked to answer two poll questions on their geographic location and whether they viewed the changes imposed by COVID-19 on their educational practice as an opportunity or challenge. These questions served both as an icebreaker and for moderators to better understand the participants and their needs. Participants were asked to reflect on the question *“What is one new strategy you have recently tried and are proud of?”* as the trigger question for the small group discussions. The question was deliberately chosen to allow participants to share their success stories and maximise peer learning, intended to enhance the sense of psychological safety.

### Small group discussions

Participants were assigned to small groups via the Zoom™ breakout room function for 30 min, during which participants were encouraged, but not pressured, to use audio and video to see one other and share experiences and educational concerns. Since participation was through open invitation, only random breakout group assignment was possible on Zoom™. One moderator facilitated the discussion and the other managed the chats. Small groups were asked to reflect on specific challenges as educators, then brainstorm and share strategies for effective teaching and learning in virtual learning environments.

### Large group reports and wrap up

The post-breakout large group discussion provided an opportunity to synthesise key points from small group conversations and generate *‘hot tips’* and take-home messages for successful virtual teaching across the continuum of HPE. Questions were posed, recorded and answered throughout the webinar using audio and chats, which allowed participants to talk in real time during the webinar and share concerns, questions, and ideas.

### Post session

After the workshop, the moderators carried out a rapid content analysis of the recorded chat and group conversations which, along with some lessons learned from running the workshop, are discussed below. All participants were sent a handout summarising these.

## Outcomes of innovation

The primary goal of the innovation was to have global and culturally diverse HPEs engage in interactive discussions about important educational challenges during the pandemic. Twelve moderators and 55 participants from a range of different health professions represented high and low resource countries: Indonesia, Malaysia, Sri Lanka, Saudi Arabia, Egypt, Myanmar, Pakistan, UK, Netherlands, Slovakia, Greece, Canada, US, Georgia, Brazil, Portugal, and Turkey. While the establishment of a psychologically safe space cannot be definitively proven, it was inferred from the enthusiastic sharing of personal experiences and stories by participants during the breakout discussions as well as suggestions offered to other educators via chat discussions. Facilitated interactive discussions supported participants in their efforts to design new educational initiatives, as suggested by the diverse topics and themes identified from participant conversations and described below.

### Overall impression

Despite the challenges brought about by the COVID-19 pandemic, participants identified significant opportunities for educational innovation as well as personal development and growth during this transition. Many reflected that HPEs can learn valuable lessons from this crisis to apply in future educational initiatives.

### Perceived challenges

**Fig. 1:**
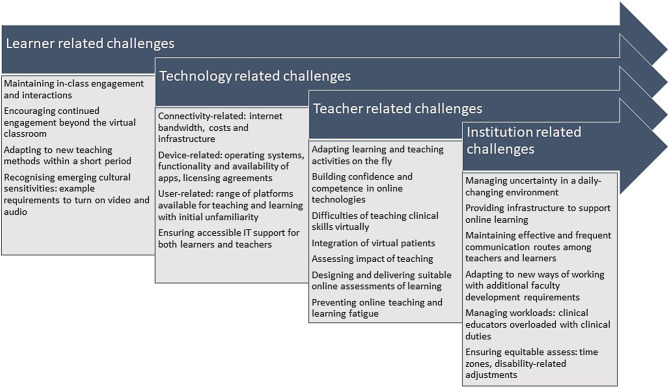
Challenges for health professions’ educators in shifting to online learning (points which came out from the discussions in the workshop)

Through the small-group discussions, a number of challenges in shifting to online learning were identified (Fig. 1). Many countries (not just low resource settings) faced problems, such as restricted or non-availability of electronic devices (e.g. laptops) for lower socioeconomic groups of students and teachers, and difficulties with network bandwidths and connections.

### Narrative themes from breakout room discussions

**Fig. 2:**
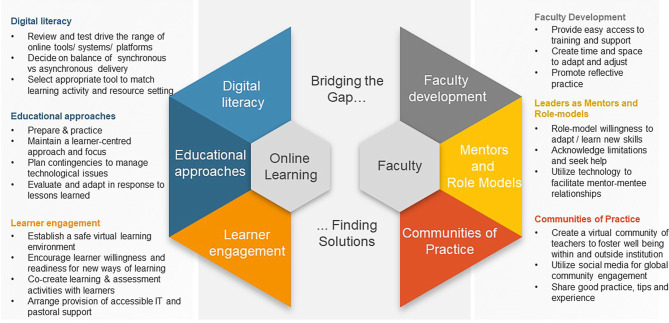
Narrative themes of enablers to change from the small group discussions in the workshop. Participants provided recommendations to overcome the challenges of the pandemic and promote effective and even innovative online experiences. These recommendations were grouped under different categories: understanding and enhancing, methods and approaches, learner-related, faculty development, mentors and role-models and learning communities

It was emphasised that educators need to maintain their authenticity, mission and vision during times of change. In countries or institutions not previously using online learning, rapid adoption was necessary. Whilst this posed challenges, it was felt to promote change and possibly innovation. Planning and delivering innovative teaching and learning opportunities for learners as well as maintaining wellbeing for themselves, learners, and colleagues were prioritised. In the small group discussions and large group reports, educators shared a range of ideas, hints, and tips, summarised in Fig. 2.

We recommend that educators who plan and participate in similar webinars pay close attention to these principles to guide future activities and support movement from ‘emergency’ online teaching and learning, towards a *‘new normal’*, where online learning is embedded into routine educational practice [3].

## Critical reflection

The process of organising this workshop offered valuable lessons to the organisers. The world café format provided opportunities for geographically dispersed HPEs to engage in conversations specific to the challenges of education during the COVID-19 crisis. A psychologically safe space is critical to foster a mindset that we are a large global community that can learn from each other. Ensuring psychological safety through icebreakers, small group discussions and a positive overall tone helps enable collegial discussions, even when the language is not a participant’s first language. For example, the icebreaker about sharing success stories prompted participants to share not only successes but also challenges. Chat comments indicated that educators from various parts of the globe face many similar challenges. Several participants also provided solutions via chat to other educators’ challenges. These interactions caused workshop leaders to infer that a safe space had been established.

Technological glitches can occur such as unexpected computer shut down, broadband connectivity, participation via smartphones, lack of video capabilities, background noise/interruption etc. Workshop organisers must ensure that all team members have assigned roles, and someone can take over from the lead presenter if needed. Participants of online learning are forgiving of technological errors and enjoy connecting with colleagues around the world. It is worth emphasising that the larger the moderating team, the more complex the planning. Leadership and followership principles must be applied diligently. Debriefing and reflection after such global events can ensure that future initiatives continue to improve in quality.

Although the primary reason for offering this workshop virtually was the outbreak of the pandemic, the format and approach are highly adaptable to a range of topics and educational needs. Global conversations add value to educators’ learning and practice and provide a safe space to share opportunities and challenges. We plan to continue organising webinars using the modified world café format, and suggest that this format could be adapted for many different purposes such as mentoring, research discussions, problem solving or journal clubs. In terms of community building, such workshops are an exemplar of the statement *“the impact is much greater than the sum of its parts.”*
